# Vocabulary Mobile Learning Application in Blended English Language Learning

**DOI:** 10.3389/fpsyg.2022.869055

**Published:** 2022-05-03

**Authors:** Petra Polakova, Blanka Klimova

**Affiliations:** Department of Applied Linguistics, Faculty of Informatics and Management, University of Hradec Králové, Hradec Králové, Czechia

**Keywords:** mobile applications, apps, English language, vocabulary learning, blended learning

## Abstract

Mobile devices and applications, which have become an integral part of our lives, are gradually used for different purposes, including learning languages in EFL classrooms. Since vocabulary plays an important role in the process of foreign language learning, the aim of this study was to explore the use of the vocabulary mobile learning application and its usefulness in blended English learning. Quantitative and qualitative approach to research was used, since the integration of both approaches created the possibility to solve complex research problem. The case study was based on the use of the developed mobile application called *Angličtina Today* the content of which corresponded to the language needs of the target group of students. The quantitative approach used a method of quasi-experiment aiming to achieve the pre-tests and post-test results of the students from the experimental and control groups. The results showed that the students facing blended learning, including mobile application in the process of language learning, achieved better results than the students exposed to the traditional, face-to-face education. In addition, the results revealed students’ overall satisfaction with the application. The main reasons for their satisfaction were improved vocabulary knowledge, ease of use, and enhanced motivation. Based on these findings from the current study, it can be argued that the vocabulary mobile learning application proved to be useful in the process of blended English language learning.

## Introduction

In recent years, blended learning (BL) has become popular in educational environment. As it combines traditional and online learning modes, the promise of BL rests on the strengths of both learning approaches. Having this in consideration, the most important aim of the BL design is to find an effective combination of different learning methods which can motivate students to participate inside and outside of the classroom environment (Neumeier, 2005; Senffner and Kepler, 2015). Senffner and Kepler (2015) pointed out that BL is flexible, scalable, and meaningful way of learning since its online component allows students to learn anytime and anywhere without being limited to groups or partners. Regarding this, the mobile technology appears to be an appropriate approach to education since smartphones and other portable Wi-Fi gadgets are related to both traditional and innovative ways of learning and are well aligned with strategic educational goals (Kukulska-Hulme, 2009). Klímová (2017) argued that characteristics such as portability, individuality, unobtrusiveness, availability, adaptability, persistence, usefulness, and usability make mobile devices an ideal language-learning tool. Moreover, the ownership of smartphones among students has grown fast which led to a great popularity of the mobile technology in education, including English as Foreign Language (EFL) learning. Mobile devices have been adapted in the traditional classroom environment and are increasingly used by English language learners (Nasab and Taki, 2016).

Since vocabulary acquisition is considered to be an essential part of language learning, vocabulary mobile learning applications (apps) have become a popular form of mobile-assisted language learning (MALL) (Klímová, 2019). Research has shown that a mobile application designed and based on students’ needs and continuously facilitated by a teacher is effective in the enhancement of students’ performance and contributes to positive learning outcomes (Mahdi, 2017; Rezaei et al., 2013; Klímová, 2019). Despite an increasing number of mobile learning applications offering a powerful learning environment, it is certain that the traditional methods of learning foreign languages are still useful. However, they need to be updated and combined with new trends in education (Mikulecký, 2019). Before the onset of coronavirus, in Slovakia, teaching and learning had been mainly based on the traditional face-to-face learning approach. The technology-based learning tools were implemented only in the case when teachers were willing to do so. After the spread of coronavirus, the traditional learning was disrupted and sudden lockdown forced teachers and students to adapt their teaching and learning. New circumstances made educational institution recognize the importance of technology-based learning tools since they appear to be effective tools for ensuring students’ education. This is especially true for mobile devices. Considering the importance of mobile technology, the present study investigated a mobile learning application called *Angličtina Today* [English Today] and its usefulness in the process of vocabulary acquisition in the EFL setting of Slovakia. The following research questions guided this research:

1. To what extent is the use of a mobile application useful for second language vocabulary acquisition?
2. What are the perceptions of EFL students of the vocabulary mobile learning application?
3. Has the use of the mobile application helped students to extend their vocabulary knowledge?

## Literature Review

It is commonly thought that vocabulary lies in the center of language learning since it is impossible to convey information without having knowledge of vocabulary (Wilkins, 1972; Nation, 2001; Zhang, 2011; Alquahtani, 2015; Gürkan, 2018). In order to increase vocabulary size, both incidental and instructional vocabulary acquisition are required. Incidental learning has been portrayed as implicit since knowledge is acquired independently of conscious attempts to learn. In other words, incidental learning is taken as it is learning without intent to learn (Nation, 2001; Eraut, 2004). According to Schmitt (2010), only ten encounters with vocabulary can lead to its sizeable gain since the repetition affects incidental vocabulary learning. According to his findings, learners who encounter an unknown word more times in informative contexts are able to demonstrate significantly larger gains in vocabulary knowledge than learners who have fewer encounters with a new word. This is in line with Soleimani et al. (2022) whose study revealed that repeated exposure to vocabulary leads to significant EFL vocabulary gains. Despite of this, there is an unknown boundary between word which has been recognized and the word which has been fully understood and learnt in the process of incidental vocabulary learning. Therefore, there is a need of some systematic approach, to ensure vocabulary acquisition which can be reached by intentional learning (Björgvinsson, 2012). It is in accordance with Zhang and Teng (2012) whose study pronounced a significant role of intentional learning. Students need to get input in forms of explanation and clear instructions and they need to practice the language they have learnt which could possibly lead to a successful output (Birkner, 2016). The role of input, information processing, and output has been gradually acknowledged in second language learning. Zhang (2009) believed that these are essential elements in foreign language acquisition, including vocabulary acquisition. Different phases of vocabulary learning can take place in different environments such as traditional school learning environment or technology-based learning environment. Combination of educational technology with traditional methods can lead to effective vocabulary acquisition and thus to achievement of balance in the learning process (Mahdi, 2017).

Poláková (2019) recommended the combination of online interaction with traditional classroom methods. She assumed that blended learning can make learning process more interesting and motivational. Moreover, the previous studies reported positive attitudes of the students toward the use of the mobile application in learning vocabulary which is in accordance with the findings of the study conducted by Lu (2008) who found out that the positive attitudes toward learning vocabulary *via* mobile phone might be influenced by its characteristics such as portability, immediacy, novelty, and legibility. With regard to the previous statement, Prensky (2005) claimed that when conducting the research regularly delivered information *via* mobile phone was manageable while the traditional manner of learning failed to arouse students’ interest in study. In other words, using mobile devices as learning tools leads to enhancement of learners’ motivation. The learning materials delivered by mobile devices are more appealing to students, which is not the case with paper-based learning materials. (Gürkan 2018), Butarbutar (2020), and Kohnke (2020) also emphasized that using mobile apps increases students’ motivation since it is more enjoyable in comparison with the traditional methods of learning. The motivation level of the participants seemed to be an important aspect of the study conducted by Ekinci (2017) since, according to him, most of the participants consider BL as motivating and entertaining because it is considered a new method of learning. This is in line with Viberg and Grönlund (2013) who found out that the perceptions of the mobile application users are extremely positive and their motivational level is high toward mobile learning.

Another benefit of using the mobile apps in the process of vocabulary acquisition is the corrective and immediate feedback. This found concordance with Liu (2008), Klímová (2019), and Klímová and Poláková (2020). The research participants of their studies claimed that feedback helps them to learn new vocabulary items and understand their meaning which helps them to improve their language skills. The other factors affecting the learning process are the ease of use and the ubiquitous character of mobile phones. They are portable devices and can be operated anywhere and anytime with no difficulties. These factors have a stimulating effect on the students and their willingness to continue using the mobile app. These findings were supported by different studies (Gafni, 2008; Ekinci, 2017; Gafni et al., 2017; Klímová and Poláková, 2020).

However, the aim of the current study is to discover the impact of the vocabulary mobile learning application on students’ range of vocabulary and to understand the students’ views on the usefulness of the vocabulary mobile learning application in the process of language learning. To the best knowledge of the researchers, no study to date has investigated the overall perception of the students of the mobile applications as learning tools in EFL context in Slovakia. In order to shed more light on this subject, the authors of this study found it an opportunity to conduct research on vocabulary acquisition through the use of BL, including mobile technology, in order to discover the impact of the vocabulary mobile learning application on students’ range of vocabulary, to define the usefulness of the application, and to understand students’ views on the usefulness of the mobile technology in the process of language acquisition.

## Materials and Methods

### Participants

A total number of 36 Slovak EFL students took part in this case study. The research participants were students with intermediate level of English of two different classes in the Secondary Vocational School of Gastronomy and Tourism, Nitra, Slovakia. The students were randomly assigned to an experimental group (*N* = 17) and a control group (*N* = 19). All the participants were students aged between 17 and 18 years. They had been learning English for 12 years on average. The English proficiency of the participants was measured by pre-test whose results indicated that there was no statistically significant difference between these two groups.

### Research Instrument

**Figure 1:**
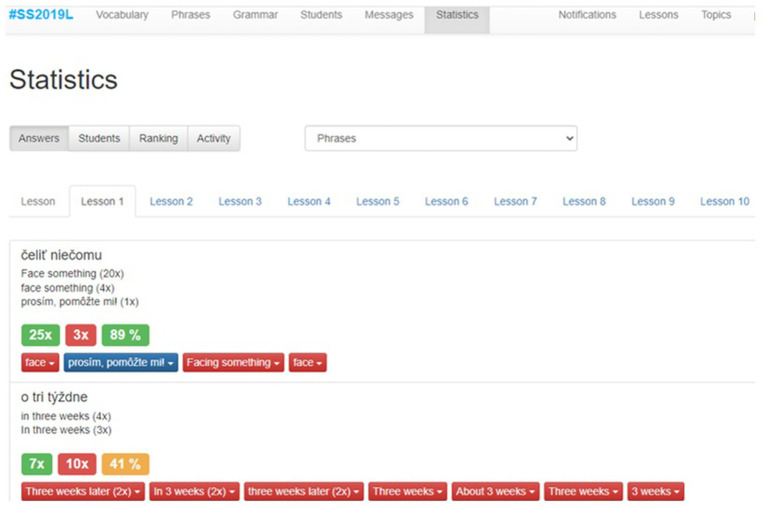
The web part of the application designed for the teachers.

The mobile application *Angličtina Today*, used as the research instrument, has two separate parts. The first application part is designed as a web interface for the teacher and it is responsible for storing information, authenticating users, efficiently collecting large data, processing, distributing messages, and responding to events. Each teacher can manage several lessons, register their students, distribute news or alerts through notifications, and respond to their comments. Using these options, the teacher can make contact with students and draw attention to the upcoming events. The key element of the web interface is the visualization of the results of all students. Based on the visualization, it is possible to evaluate each student separately, to compare the results between several study courses, or to modify the study plan (Berger et al., 2019). Figure 1 portrays the web part of the app designed for teachers.

**Figure 2:**
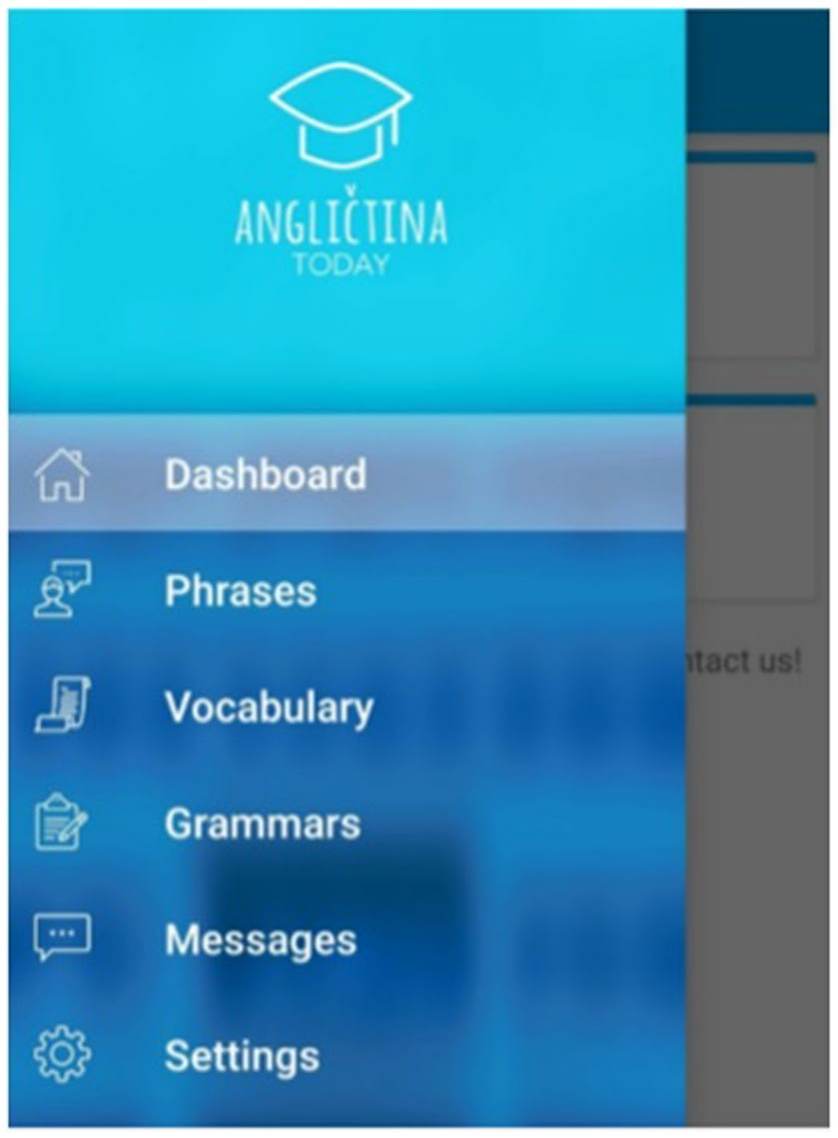
An overview of the students’ application.

The second application part is presented with a mobile application for students. Through a mobile application, the students are enrolled into a specific course to study and test available vocabulary and phrases. For each phrase or vocabulary, students can get a translation, while using TextToSpeech technology, as well as pronunciation. The application also enables immediate communication with the teacher. At the same time, the application collects all user data and distributes it to the server part to be evaluated by teacher. The students are advised by means of notifications, e.g., to study a certain lesson (Berger et al., *ibid.*). An overview of the students’ mobile app is shown in Figure 2.

### Procedure

This case study was conducted in the winter semester of 2021. The intervention, which lasted 10 weeks, was carried out to investigate the usefulness of the mobile application which was defined by an improved language proficiency and general overall satisfaction of the users with the application. During the first session, both experimental and control groups were given a pre-test in order to prove the same level of language proficiency. Consequently, students from the experimental group were facing BL approach. Firstly, they were introduced to new vocabulary in the traditional classroom environment; secondly, mobile application intervention was used to process the information; and thirdly, new vocabulary gained was used in context. It was done through different activities such as role-plays, discussions, or debates on the corresponding topics, in the traditional school environment. Meanwhile, students from the control group were facing traditional approach to learning. At the end of the mobile application treatment, the post-test was given to the participants of both groups. Moreover, students from the experimental group were required to answer minute papers’ questions after completing each online lesson, to fill the questionnaire and participate in the virtual interview.

### Data Collection and Analysis

Based on the concept of Yin’s (2009) case study, different quantitative and qualitative methods were used in order to collect and analyze data. Aiming to compare the proficiency level of the research participants before and after the mobile application intervention, standardized multiple pre-test and post-test were used in the quasi-experimental phase of the study. The test results were calculated using IBM SPSS Statistics 26 software. After the vocabulary mobile learning application treatment, the questionnaire survey was conducted as a complementary method to quasi-experiment. The results obtained from the quantitative data collection were analyzed by descriptive statistics.

**Figure 3:**
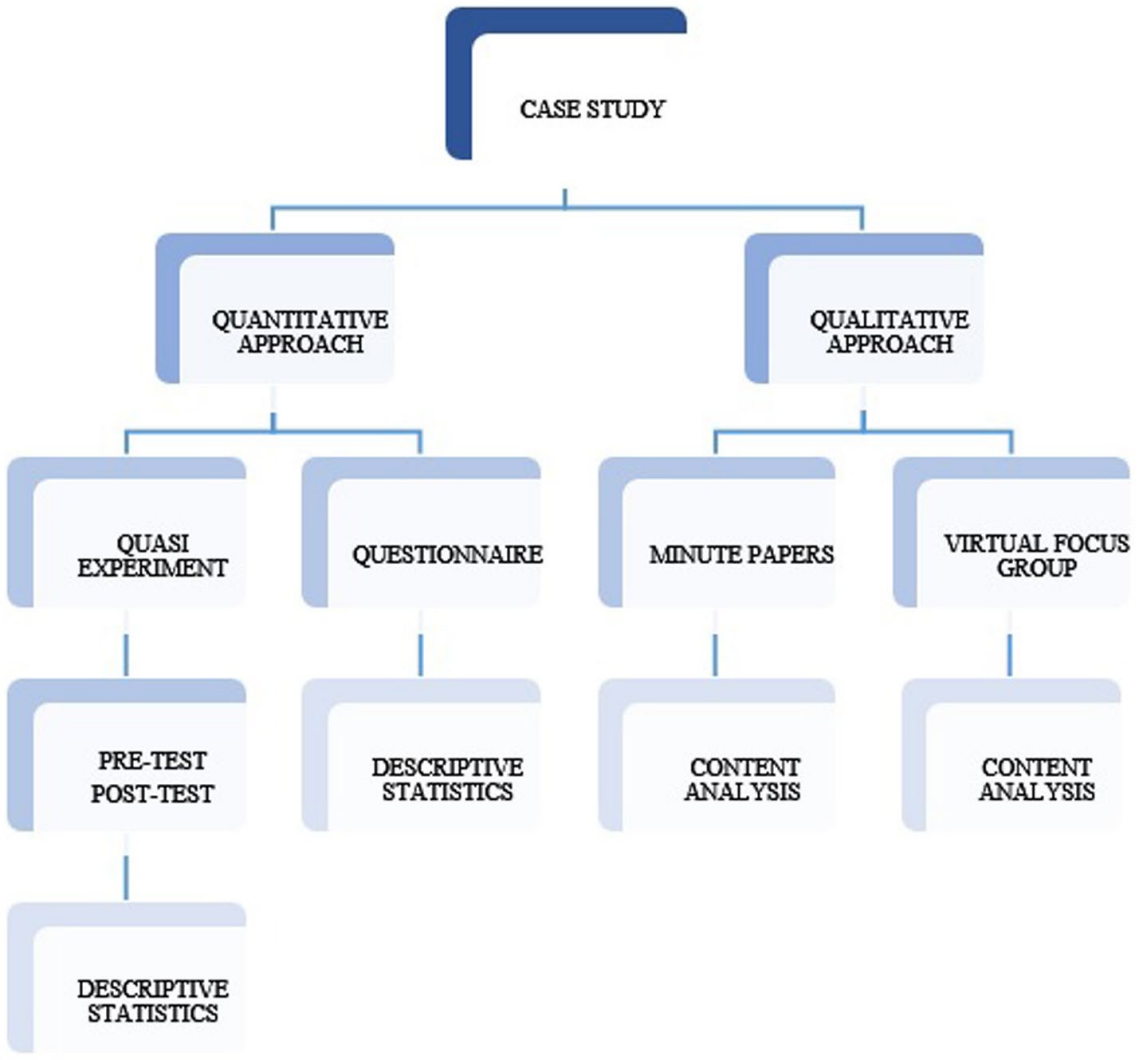
Research design.

The qualitative data were collected to understand the students’ perceptions of the mobile vocabulary learning application. Students were provided minute papers while using the mobile application. Despite the fact that minute papers represent a very commonly used classroom assessment technique, in the current study, it was used as the method of qualitative data collection. Moreover, virtual focus group was organized after the mobile application treatment. The ZOOM platform was used in the present study to interview the research participant. The results gained from the qualitative methods were analyzed by categorization and coding. For better understanding, the research design is provided in Figure 3.

## Results

The aim of this study was to obtain information on the usefulness of the mobile application. On the basis of the research analyses, it may be stated that the results obtained from the quasi-experiment, questionnaire, minute papers, and virtual focus group have been in agreement. There were many results to comment on, but those which could be mutually compared were specifically m-learners’ performance, m-learners’ satisfaction, m-learners’ motivation, content quality, and ease of mobile application use. The comparison and the interpretation of the research results are provided below.

### Quantitative Approach to the Research

The group N consisting of 36 students was divided into two independent groups. The experimental group, the size of which was *n*_1_ = 17, used the vocabulary mobile learning application in the process of language learning. The control group, whose size was *n*_2_ = 19, did not use the mobile application and was taught by the use of the traditional methods. Students’ results were measured by the pre-test and post-test and their values were recorded. The results were calculated using IBM SPSS Statistics 26 software. A significance level of 5% was considered when performing the tests.

**Figure 4:**
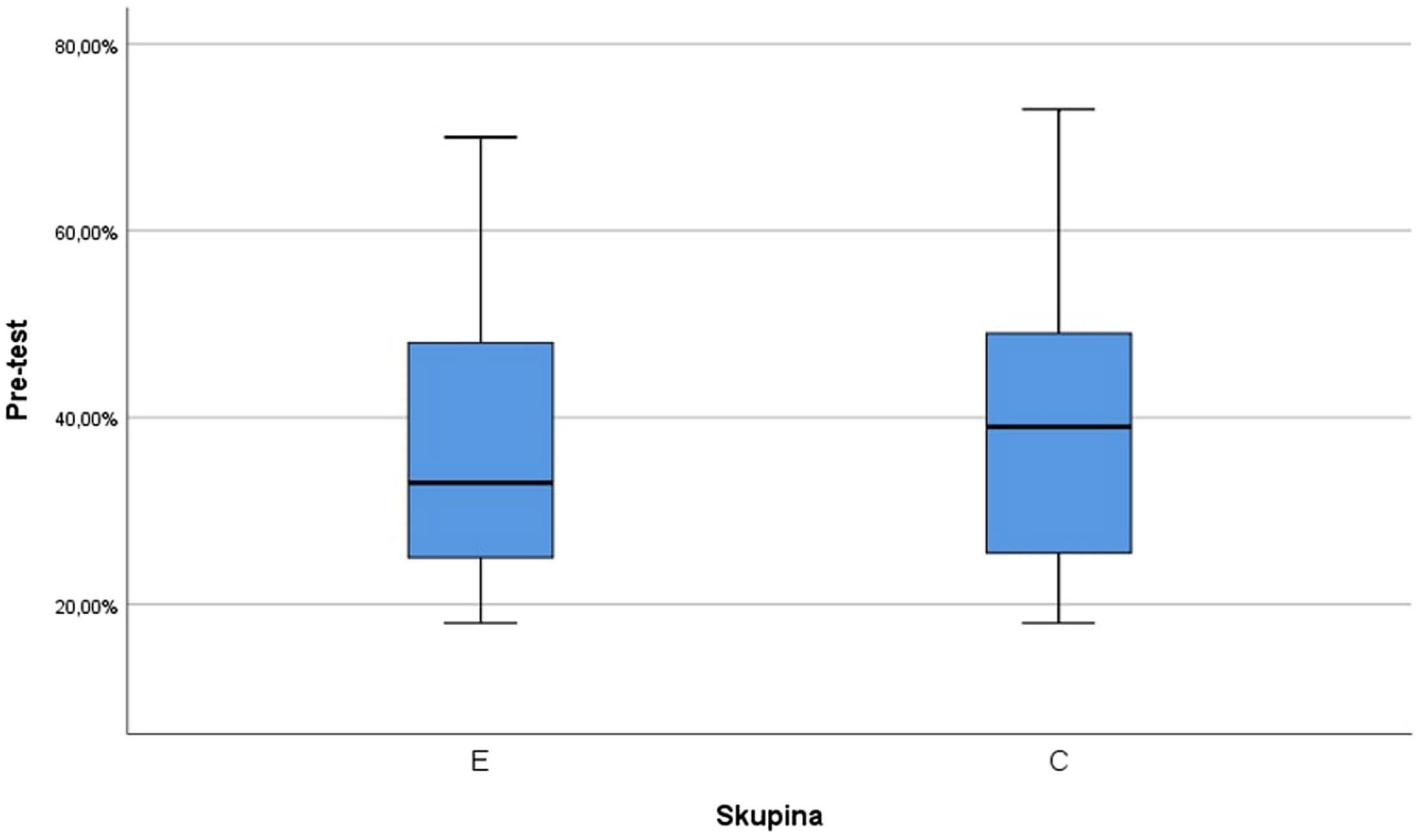
Box diagram of pre-test results for the experimental (E) and control (C) groups.

In order to assess whether the students’ knowledge of the subject, specifically English language, did not differ in the groups at the beginning of the study, the pre-test was revealed. The results divided according to both groups are shown in Table 1 in which the success of students is given as percentages and Figure 4.

**Table 1:** Descriptive statistics of the experimental (E) and control (C) groups in the pre-test.

Group	Mean	Std. deviation	Median	Minimum	Maximum
E (n_1_ = 17)	38.18%	16.88%	33.0%	18.00%	70.00%
C (n_2_ = 19)	39.68%	16.83%	39.0%	18.00%	73.00%

The Shapiro–Wilk test did not reject the hypothesis that both the selection in the E group (value of *p* 0.061) and the selection in the C group (value of *p* 0.271) came from a normal division of the students. The variances in both groups can be considered identical based on Levene’s test (*F* = 0.024, value of *p* 0.877). To compare the mean values of the pre-test results, a *t*-test could be used for two independent samples with the same variances (*t* = −0.268, value of p 0.790), according to which the hypothesis of equality of mean values was not rejected. It can be assumed that at the beginning of the study, i.e., at the time when the pre-test was given, the average results of students in both groups did not significantly differ. Furthermore, the following research hypothesis was stated as:

*H*: The students using the vocabulary mobile learning application achieve significantly higher learning outcomes that the students not using the application.

**Figure 5:**
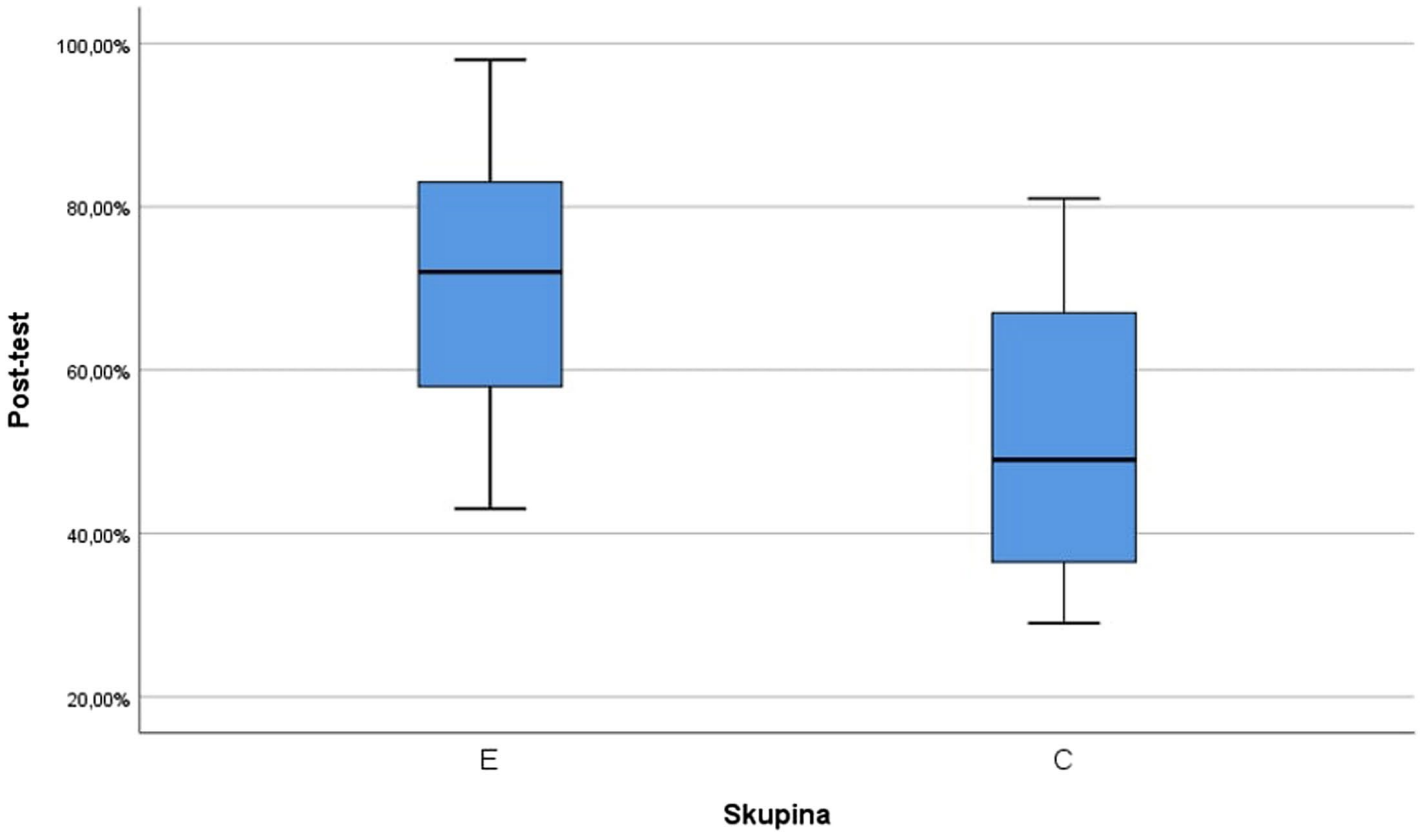
Box diagram of post-test results for the experimental (E) and control (C) groups, 10,000%.

The box diagram of the post-test results of both groups is shown in Figure 5. Based on this, it can be concluded that the students from the research group (E) achieved higher results than the students from the control group (C), as expected. However, we concluded this observation by calculation. Descriptive statistics of the post-test results are provided in Table 2.

**Table 2:** Descriptive statistics of the experimental (E) and control (C) groups in the post-test.

Group	Mean	Std. deviation	Median	Minimum	Maximum
E (n_1_ = 17)	71.47%	16.45%	72.0%	43.00%	98.00%
C (n_2_ = 19)	52.26%	17.33%	49.0%	29.00%	81.00%

The Shapiro–Wilk test did not reject the hypothesis that both the selection in the experimental group (value of *p* 0.598) and the selection in the control group (value of *p* 0.110) came from a normal division of the students. The variances in both groups can be considered identical based on Levene’s test (*F* = 0.001, value of *p* 0.976). To compare the mean values of the post-test results, a *t*-test could be used for two independent samples with the same variances (*t* = 3,400, value of *p* 0,002), according to which the hypothesis of equality of mean values was rejected. Based on this test, it can be stated that at the level of significance of 5%, there is a statistically significant difference between the results of the post-test for the experimental and control groups. After comparison of the found estimates of the mean values of the classification in both groups, hypothesis H can be accepted.

Furthermore, the following consideration assessed whether there was a statistically significant improvement of individuals in the experimental group. Students’ results in the pre-test and post-test (*t* = 13.949, value of *p* < 0.001) were compared by using a paired t-test. Based on the results of this test, it can be assumed, at the level of significance of 5%, that there is a significant difference between the results of individual students in the pre-test and post-test. As the results obtained in the post-test are higher (see the line E in the Tables 1, 2), it can be stated that the use of a mobile application leads to a significant improvement in the learning of languages.

For a more comprehensive assessment, the ANOVA can be performed with repeated measurements using the time/test factor, whose values are pre-test and post-test, and the group factor, whose values are experimental and control groups, as two independent factors (Hendl, 2005). Thanks to the method of measurement, where the measurement was repeated on the selected research sample/students, the time/test factor can be considered to be a factor with a within-subject effect, and a group factor that divides the population can be considered to be a factor expressing an intergroup effect (between-subject effect). The Mauchly test was used to verify sphericity. ANOVA for repeated measures confirmed the effect of time/test (*F* = (1.34) = 253.515; value of *p* < 0.001 and correlation ratio 0.882), thus, an increase in the average score in the post-test. The group influence was not statistically significant (*F* = (1.34) = 2.636, value of *p* 0.114 and correlation ratio 0.072), i.e., it does not matter to which group, experimental or control, the student belongs. The calculations also show that the interaction between the group factor and the time/test factor (*F*(1.34) = 51.697, value of *p* < 0.001 and correlation ratio 0.603) is statistically significant, which with respect to the values, given in Tables 1, 2 means that students in the experimental group have a significantly higher average success rate in the post-test than other students. Regarding this, hypothesis H was supported.

### Qualitative Approach to the Research

#### M-Learners’ Performance

The students’ perception of the vocabulary enhancement was examined by using the questionnaire and minute papers. The findings showed that a high percentage of the students (82%) perceived an improvement in vocabulary knowledge. In addition, the mobile application users (57%) stated that their vocabulary retention had been better, in other words, they remembered the vocabulary and phrases provided in the mobile app and were able to use them in the conversation, in the phase of output. Furthermore, the students stated that they had been able to express themselves better and more fluently after mobile application intervention (71%). Based on these findings, it can be claimed that the use of the mobile application helped students to improve their language skills. Moreover, an improvement was also perceived by the research participants.

#### M-Learners’ Satisfaction

The questionnaire results showed that 82% of the students had been satisfied with the mobile application and would recommend it to others. This is in accordance with the minute papers and virtual focus group findings, which revealed that the students had especially appreciated an easy access to the study materials, the clear arrangement, and fast learning. Most of the students (64%) agreed that learning and practicing vocabulary through the mobile application had suited them better than learning and revising new words through the book. However, many of them also pointed out that the combination of the traditional learning and innovative methods had been an interesting and satisfying form of learning; therefore, they (64%) would like to continue using the mobile app in the future. The results indicate the students’ satisfaction with the mobile application.

#### M-Learners’ Motivation

The increase of the students’ motivation was examined through the questionnaire, minute papers, and virtual focus group. The results revealed from the questionnaire showed that students (71%) had felt motivated by using the mobile application in the process of education. Learning was perceived as more fun and less stressful (100%). This information was also confirmed in the minute papers since many students added that their motivation was increased thanks to better learning outcomes, which were verified in the form of the additional tests. Based on the data obtained from the focus group, it can be stated that the above-mentioned tests were perceived as extrinsic motivation for students to use the mobile application. Nevertheless, such motivation was not perceived negatively by students. At the same time, mobile application users’ intrinsic motivation was to increase their language skills. Regarding the findings from the various methods, the results demonstrate that the mobile application has a motivating effect on students.

#### Mobile Application’s Functions

When comparing the data obtained from the questionnaire, the minute papers and the virtual focus group, it was found that the results of these methods matched. For a better understanding, the information provided in the mobile application was considered clear, readable, and easy to absorb (100%). The corrective feedback function was perceived as the biggest advantage of the mobile application (100%), as it helped students to learn and understand vocabulary faster. In addition, according to the students’ opinion, the corrective feedback helped them to improve and extend their vocabulary, which was also claimed by the results obtained from the post-test. On the contrary, the mobile application notifications and the pronunciation verification were considered to be the shortcomings of the mobile application (71%) since they did not work for many students during the research period. It was also confirmed that the students were not satisfied with the fact that the application could not be used offline. It can be stated that despite the benefits of the mobile application which helped students to improve their language skills, *Angličtina Today* proved to have certain shortcomings that should be eliminated in order to make it more effective in the process of language learning.

#### Ease of Use

The findings revealed from the different research methods were in concordance, since they confirmed that the vocabulary mobile learning application was easy to use (100%). Students stated that using the mobile app was much easier to use in order to revise the vocabulary than using the textbooks. The mobile application users appreciated the possibility to use the mobile application anytime and anywhere, its easy manipulation, and the time-saving work with it. Therefore, it can be stated that *Angličtina Today* was perceived to be a user-friendly mobile application.

## Discussion

The aim of the current research was to explore the usefulness of the mobile application defined by an improved language proficiency and also general overall satisfaction of the users with the app. The results from this study offer some key findings:

First, the questionnaire, minute papers, and virtual focus group revealed the following positive perceptions of the mobile application users:

– Vocabulary knowledge was improved after the mobile application treatment.
– Vocabulary retention was better after using the mobile application.
– Students’ ability to express themselves more fluently was enhanced.
– Students would recommend *Angličtina Today* to others since the easy access, clear arrangement, and fast learning were appreciated by them.
– Learning vocabulary through the mobile application was found to be more practical than using the textbook for the same purpose.
– Students’ motivation was increased thanks to the better learning outcomes.
– Learning was perceived as more fun and less stressful.
– The mobile application was considered clear, readable, and easy to absorb.
– The corrective feedback function helped students to improve their vocabulary.
– The vocabulary mobile learning application was easy to use.
– The ubiquitous character, easy manipulation, and time-saving work with the mobile application were appreciated.

These findings accord with those of Gafni et al. (2017) which revealed that using vocabulary mobile learning application has a stimulating effect on the process of learning and contributes to the willingness to continue using the application and to recommend it to others. These findings also support those of Kacetl and Klímová (2019) who indicated that the key benefits of MALL are the enhancement of the learner’s cognitive capacity, the learner’s motivation to study in both formal and informal settings, the learner’s autonomy and confidence, as well as the promotion of personalized learning, and helping low-achieving students to reach their study goals. These findings also re-echo Nuareni’s (2020) claim that the majority of the students has positive perception on the usage of MALL in learning English language. These findings are also in line with the most recent study by Jeong (2022), who claim that MALL helped his students with access to learning contents, created flexible and self-directed learning environment, better interaction, and improved self-efficacy in English learning performance.

Second, the implementation of the vocabulary mobile learning application was effective because the students from the experimental group reached better results than the students from the control group. Moreover, an enhancement of the language skills was also perceived by the mobile application users. This is in line with the results of several other studies that implemented MALL in the process of education when conducting research. For instance, Alqahtani and Mohhamad (2015) claimed that using the mobile learning application enhanced students’ performance as well as their satisfaction. Similar results were found by Lu (2008)who stated that the participants from the experimental group identified more vocabulary than the group learning vocabulary through the printed materials. The same findings were revealed by Palalas (2011) and Gharehblagh and Nasri (2020) who indicated that MALL helped to improve the vocabulary knowledge of EFL students.

Overall, the results obtained from the different quantitative and qualitative research methods proved a high level of the mobile application usefulness. This claim is based on the fact that using mobile application in the process of education helped students to reach better results in the post-test, helped to improve students’ vocabulary and the ability to express themselves more fluently, proved to be user-friendly, and enhanced students’ motivation to learn. Nevertheless, as Klimova (2021) states, teachers have to guide their students in the use of such an app to make the learning process effective and meaningful.

This study helps to understand the perceived advantages and disadvantages of using vocabulary mobile learning application in blended foreign language learning. It turned out that most of the participants found the mobile application beneficial additional learning tool since it helped them to improve their language skills and made learning more fun and less stressful. The obtained research findings can help foreign language teachers to understand the benefits of combining mobile learning with traditional face-to-face learning. It can motivate them to innovate their teaching and lose the fear of using technology due to its seemingly disruptive nature. Furthermore, it might motivate foreign language students to use their mobile devices as the learning tool. Moreover, the findings can assist developers of vocabulary mobile learning applications since students’ preferences are provided in the present study.

## Implications

In view of the results of this study, some pedagogical recommendations are suggested to EFL instructors. Teachers should be the role models for their students and **teach them how to use mobile phones in the process of language learning**. Despite the fact that students use their mobile phones on a daily basis, they might not know how to use them as learning tools. Therefore, they need the guidance of a teacher.

It is sufficient for MALL to be implemented only in one of the learning phases, preferably during the information processing phase. Students can be introduced new vocabulary in the classroom environment through the traditional methods of learning and, subsequently, **the new vocabulary can be revised through a mobile application, outside the school environment**. The last phase of output should take place in the classroom, where the various activities can be used to verify students’ vocabulary knowledge.

When revealing the mobile lessons, it is important to provide learning materials that are in concordance with the curriculum taught in the classroom. **The information provided should be well organized, readable, and easy to absorb.**

It is not necessary to provide a large amount of vocabulary in each mobile lesson. However, **it is recommended to add new vocabulary regularly**, preferably after each face-to-face lesson.

Teachers should regularly **send notifications** and remind their students of new material or testing. If mobile application notifications do not work, it is necessary to look for new solutions to inform students about these events.

**It is important to carry out a formative assessment**, which makes it possible to monitor students’ progress. The results obtained from the assessment can help teachers to understand the needs of the students and possibly change the learning plans. Formative assessment can also be a motivation for students to use the mobile application; moreover, students are provided with feedback on their learning performance.

It is essential for teachers to **ask for feedback from the students** and understand their perception of the use of new technologies in the learning process. It can help teachers to improve their teaching and, what is more, students value being asked for their opinion since they feel being recognized when they are given space to express their views. Such an approach can help teachers to create a positive school climate and thus improve the learning process.

It is recommended to **give students a free choice** and let them decide whether they want to use the mobile technologies in the process of learning or prefer traditional learning through textbooks. Forcing students to use the mobile technologies could provoke resistance to learning. Students must not be punished for their decisions.

Furthermore, it would be suitable to **raise other teachers’ awareness** of the implementation of the mobile technologies in the process of the language learning which can help students to improve their language skills. The older generation of teachers often resist the use of innovative trends, but nowadays, the implementation of the technology in education seems to be essential. The COVID-19 pandemic clarified the importance of the technologies as traditional learning suddenly switched into distance learning. This could not have worked without the inclusion of the technology in the process of learning.

## Limitations

Naturally, there are certain limitations to be considered in this study. To start with, the research sample size was small; therefore, it was difficult to get more statistically significant results and generalize the study. Additionally, the time the secondary school students experienced the mobile application was relatively short. A longer period may have provided an additional and deeper insight. Furthermore, there was no space to examine the vocabulary retention perceived by the students in the follow-up period. However, this fact was confirmed by different studies whose results were in line with the findings of the present research.

## Data Availability Statement

The raw data supporting the conclusions of this article will be made available by the authors, without undue reservation.

## Author Contributions

All authors listed have made a substantial, direct, and intellectual contribution to the work and approved it for publication.

## Funding

The paper is supported by the project Excellence (2202/2022) at the Faculty of Informatics and Management, University of Hradec Králové, Czechia.

## Conflict of Interest

The authors declare that the research was conducted in the absence of any commercial or financial relationships that could be construed as a potential conflict of interest.

## Publisher’s Note

All claims expressed in this article are solely those of the authors and do not necessarily represent those of their affiliated organizations, or those of the publisher, the editors and the reviewers. Any product that may be evaluated in this article, or claim that may be made by its manufacturer, is not guaranteed or endorsed by the publisher.
